# The association of serum procalcitonin and high-sensitivity C-reactive protein with pneumonia in elderly multimorbid patients with respiratory symptoms: retrospective cohort study

**DOI:** 10.1186/s12877-016-0192-7

**Published:** 2016-01-15

**Authors:** Antonio Nouvenne, Andrea Ticinesi, Giuseppina Folesani, Nicoletta Cerundolo, Beatrice Prati, Ilaria Morelli, Loredana Guida, Fulvio Lauretani, Marcello Maggio, Rosalia Aloe, Giuseppe Lippi, Tiziana Meschi

**Affiliations:** Department of Clinical and Experimental Medicine, University of Parma, Parma, Italy; Internal Medicine and Critical Subacute Care Unit, Geriatric-Rehabilitation Department, Parma University Hospital, Parma, Italy; INAIL-CERT Research Center at University of Parma, Parma, Italy; Laboratory of Clinical Chemistry and Hematology, Parma University Hospital, Parma, Italy

**Keywords:** Procalcitonin, High-sensitivity C-reactive protein, Pneumonia, Multimorbid elderly

## Abstract

**Background:**

Serum procalcitonin and high-sensitivity C-reactive protein (hs-CRP) elevations have been associated with pneumonia in adults. Our aim was to establish their diagnostic usefulness in a cohort of hospitalized multimorbid patients ≥65 years old admitted to hospital with acute respiratory symptoms.

**Methods:**

With a retrospective cohort study design, all multimorbid patients ≥65 years-old with acute respiratory symptoms admitted to an internal medicine hospital ward in Italy from January to August 2013 were evaluated. Pneumonia diagnosis, comorbidities expressed through Cumulative Illness Rating Scale (CIRS), setting of living, length of stay, serum hs-CRP and procalcitonin at admission were collected for each patient. Data were analyzed with Mann-Whitney’s *U* test and multivariate Cox logistic regression analysis. A Receiver Operating Characteristic (ROC) curve was used to verify each biomarker’s association with pneumonia diagnosis.

**Results:**

Four hundred fifty five patients (227 M) were included in the study, of whom 239 with pneumonia (138 M, mean age 80 ± 13) and 216 without pneumonia (89 M, mean age 80 ± 14). After adjustment for age and sex, median levels of hs-CRP were significantly higher in patients with pneumonia (116 mg/L, IQR 46.5–179.0, vs 22.5 mg/dl, IQR 6.9–84.4, *p* < 0.0001), while procalcitonin median levels were not (0.22 ng/ml IQR 0.12–0.87, vs 0.15 ng/ml, IQR 0.10–0.35, *p* = 0.08). The ROC analysis showed that, unlike procalcitonin, hs-CRP values were predictive of pneumonia (AUC 0.76, 95 % CI 0.72–0.79, *p* < 0.0001, cut-off value 61 mg/L), even after adjustment for possible confounders including nursing home residence and dementia. Serum hs-CRP levels >61 mg/L were independently associated with a 3.59-fold increased risk of pneumonia (OR 3.59, 95 % CI 2.35–5.48, *p* < 0.0001).

**Conclusion:**

In elderly multimorbid patients who require hospital admission for respiratory symptoms, serum hs-CRP testing seems to be more useful than procalcitonin for guiding the diagnostic process when clinical suspicion of pneumonia is present. Procalcitonin testing might hence be not recommended in this setting.

## Background

Community-acquired pneumonia (CAP) and healthcare-related pneumonia (HCAP) are highly prevalent in the multimorbid elderly population, accounting also for a high number of hospital admissions. In subjects aged ≥65 years, CAP incidence of 14 per 1000 person-years has been reported [1]. About 30 % of patients hospitalized with pneumonia in industrialized countries live in nursing homes, and thus these cases may be classified as HCAPs [2].

The diagnosis of pneumonia in elderly patients, whatever the healthcare setting of provenience, is often challenging. Signs and symptoms, including dyspnea, may be vague or non-diagnostic, chest X-ray (CXR) findings may be inconclusive and comorbidities may act as confounders [3]. Therefore, the use of biomarkers of inflammation or infection, such as procalcitonin and C-reactive protein (CRP), has been proposed as a guide in the diagnostic process [4].

The role of these two biomarkers in acute-care settings has been extensively studied in recent years. The usefulness of procalcitonin in patients with unclear respiratory symptoms has been particularly emphasized [5–7]. This biomarker has been demonstrated as useful for differentiating pneumonia from other acute clinical conditions [8–11] and for establishing the prognosis [12–16]. However, some studies carried out in different settings, such as acute-care wards [17], intensive care units (ICUs) [18] and primary care [19, 20], questioned these assumptions.

CRP is a well-established biomarker in many clinical settings, but has been traditionally considered not enough specific to be a useful guide in the diagnostic process of pneumonia. In fact, virtually all infective, autoimmune, ischemic and neoplastic diseases can contribute to increase serum CRP values. Nevertheless, some studies have confirmed that it may have a good performance in defining pneumonia diagnosis and severity [21, 22].

Very few studies assessing the role of CRP and procalcitonin in the diagnostic algorithms of pneumonia have been focused on multimorbid elderly patients, with controversial results [7, 17]. These patients, although representing the large majority of cases of pneumonia admitted to acute-care hospital wards, are often excluded from clinical studies due to the possible presence of multiple confounders. However, this is the setting where efficient biomarkers are mostly needed, considering also the possible pitfalls of traditional diagnostic examinations, such as CXR.

Therefore, the aim of the present retrospective observational study is to evaluate the role of procalcitonin and high-sensitivity C-reactive protein (hs-CRP) in establishing the diagnosis of pneumonia in a cohort of multimorbid patients over 65 years old admitted with respiratory symptoms to an acute-care hospital ward.

## Methods

### Patients and methods

A retrospective evaluation on clinical records of all patients admitted from January 1st to August 31st 2013 to the Internal Medicine and Critical Subacute Care Unit of Parma University Hospital, Italy, was performed. This unit mainly admits elderly frail multimorbid patients from Emergency Department. To exclude possible cases of hospital-acquired pneumonia (HAP), only patients with no hospital admission in the previous 30 days were included in the study. Additional inclusion criteria were age ≥65 years old, presence of respiratory symptoms (dyspnea, cough, sputum, hemoptysis, pleuritic pain) as main reason of admission, absence of a well-defined terminal condition with a survival prognosis <30 days and presence of at least one of feature of frailty (grip strength lower than 18 kg, reduced gait speed, forced bed rest, lack of autonomy in activities of daily living, >5 % weight loss in the previous 6 months) or a Rockwood Clinical Frailty Scale score ≥5 [23, 24].

### Data collection

For each eligible patient, age, sex, setting of living (community vs nursing home), length of stay in our unit, pneumonia status, main diagnosis, chronic comorbidities, CXR/chest Computed Tomography (CT) results and serum levels of procalcitonin and hs-CRP at admission were collected. The diagnosis of pneumonia was set through a positive chest imaging technique, usually CXR, performed in every patient admitted to our unit with respiratory symptoms, while chest CT scan was performed in case of inconclusive CXR findings. Multimorbidity was measured with the number of diagnoses and with Cumulative Illness Rating Scale (CIRS) Comorbidity Score [25]. This index is validated in scientific literature [26] and based on the assignment of a score (from 0 to 4), equivalent to disease severity, to each of 14 items that represent possible organs of systems that can be affected by a chronic disease. CIRS Comorbidity Score is defined as the sum of all scores assigned to 14 items.

hs-CRP was measured with a reference immunonephelometric assay on BN system (Siemens Healthcare, Tarrytown, New York). The diagnostic cut-off of hs-CRP was set by manufacturer at 5 mg/L, with a total imprecision lower than 5 %. Procalcitonin was measured with a validated immunoassay (B.R.A.H.M.S. Kryptor system, BRAHMS, Hennigsdorf, Germany). The functional sensitivity of this method is 0.04 ng/mL and the total imprecision is comprised between 0.7 and 8.3 %.

According to the aims of the study, patients with respiratory symptoms and a final diagnosis of pneumonia and those with respiratory symptoms without pneumonia were analyzed separately. Serum levels of hs-CRP and procalcitonin were routinely measured within 12 h from admission to Internal Medicine and Critical Subacute Care Unit during the period of the study. Patients with missing data or blood tests performed after 12 h from admission were excluded from the final analysis.

### Statistical analysis and data management

Data were analyzed using SPSS v.22.0 software (IBM, Armonk, New York, USA) and SAS v.8.2 (SAS Institute Inc., Cary, North Carolina, USA). When data distribution deviated significantly from normality, continuous data were reported as median values and interquartile range (IQR). Subjects with and without pneumonia were compared using Mann–Whitney *U* test for continuous variables and chi-square (*χ*^2^) test for categorical variables, adjusted for age and sex. Receiver Operating Characteristic (ROC) curves were used to evaluate the sensitivity and specificity of procalcitonin and hs-CRP vs pneumonia diagnosis. Cut-off values were reported with the area under the curve (AUC) being given with its 95 % confidence interval (CI). The cut-off value was calculated with the Youden index. Univariate and multivariate analysis with Cox regression models were then applied to identify parameters associated with pneumonia diagnosis. Confounders were selected among those parameters that were significantly different between subjects with and without pneumonia at univariate analysis.

Additional subgroup analyses were also performed according to CAP/HCAP categorization and to positivity or negativity of CXR, in order to verify the diagnostic accuracy of procalcitonin and hs-CRP in these situations.

All *p*-values were two-tailed and were considered significant for *p* < 0.05.

### Ethics, consent and permissions

The study protocol was approved by local ethics committee (Comitato Etico per Parma), ID n. 39655. All phases of the study were carried out in conformity with the principles expressed in the Declaration of Helsinki. Written informed consent was obtained according to Italian law.

## Results

The overall number of admissions performed from the Emergency Department in Internal Medicine and Critical Subacute Care Unit from January to August 2013 was 1567. On overall, 1115 patients were excluded for not satisfying inclusion criteria. Namely, 744 patients were excluded since respiratory symptoms were not the main cause of admission, 174 for age (i.e., being ≤65 years old), 101 for hospitalization in the 30-day period before admission and 93 for other reasons (terminality, lack of “frailty” criteria, good performance status, missing data about hs-CRP and procalcitonin, blood tests performed after 12 h from admission). Thus, the final study population included 455 patients (227 M, 228 F).

The number of patients discharged with a main diagnosis of pneumonia (P+ group) was 239 (138 M, 101 F, mean age 80 ± 13 years). The other 216 patients (89 M, 127 F, mean age 80 ± 14) had not pneumonia despite admission for respiratory symptoms (P- group). The main characteristics of these two groups and an age- and sex-adjusted comparison are reported in Table 1.

**Table 1:** Comparison of characteristics of patients with pneumonia (*n* = 239) and patients without pneumonia (*n* = 216) admitted for respiratory symptoms

	Patients with pneumonia (*n* = 239)	Patients without pneumonia (*n* = 216)	*p* value, age and sex-adjusted
Age, mean ± standard deviation (years)	80 ± 14	82 ± 10	0.22
Men, number (%)	138 (58)	89 (41)	0.006
CIRS Comorbidity Score, median [IQR]	14 [10–17]	14 [10–18]	0.51
Number of comorbidities, mean ± standard deviation	3.9 ± 1.5	4.0 ± 1.3	0.75
Nursing home residents, number (%)	46 (19)	24 (11)	0.004
Ward length of stay, mean ± standard deviation (days)	4.9 ± 3.1	3.8 ± 2.1	<0.001
Inhospital death, number (%)	19 (8)	16 (7)	0.96
hs-CRP, median [IQR] (mg/L, normal range 0–5)	116.0 [46.5–179.0]	22.5 [6.9–84.4]	<0.001
Procalcitonin, median [IQR] (ng/ml, normal range 0–0.50)	0.22 [0.12–0.87]	0.15 [0.10–0.35]	0.08
Dementia, number (%)	100 (42)	54 (25)	<0.001
Stroke, number (%)	57 (24)	39 (18)	0.11
Cancer, number (%)	53 (22)	41 (19)	0.56
Liver disease, number (%)	31 (13)	21 (10)	0.53

**Fig. 1:**
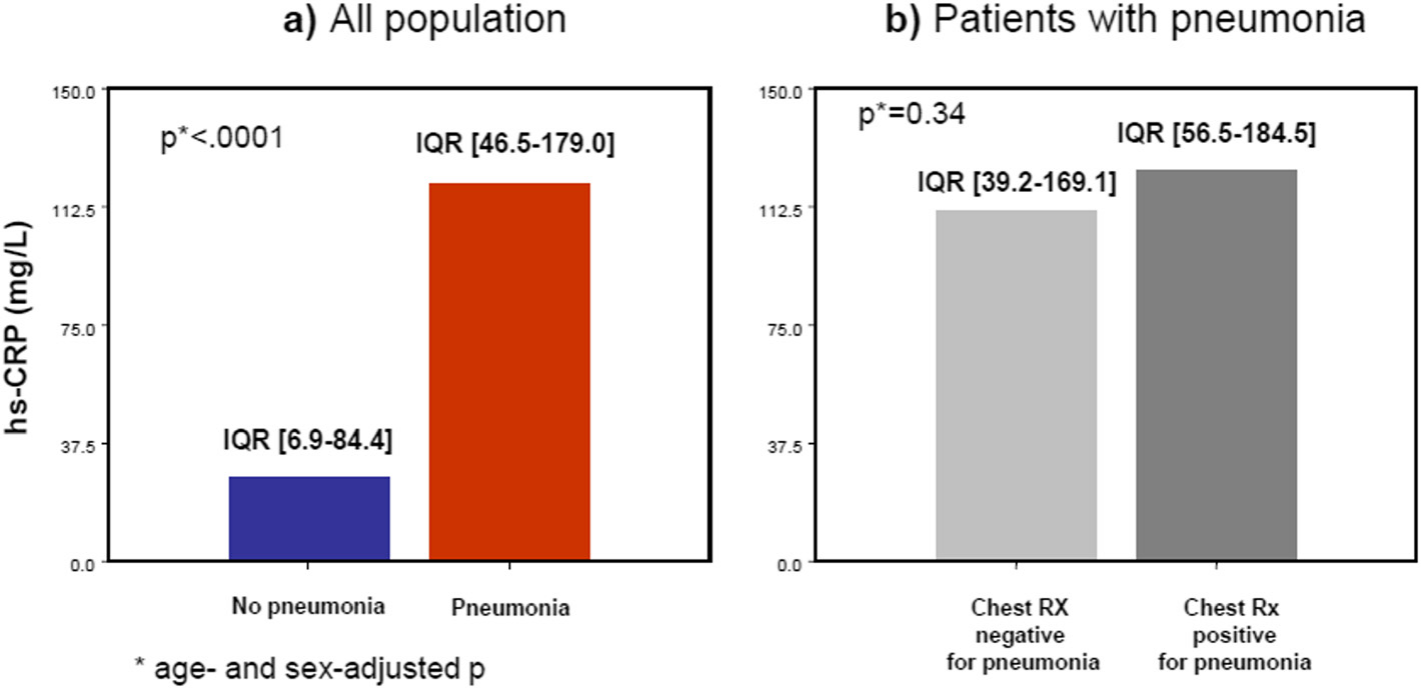
Serum high-sensitivity C-reactive protein (hs-CRP) as marker of pneumonia. Panel **a**) Comparison of hs-CRP values in patients with (*n* = 239) and patients without pneumonia (*n* = 216). Panel **b**) Comparison of serum hs-CRP values between patients with pneumonia and a diagnostic chest X-ray (*n* = 125) and patients with pneumonia and a non-diagnostic chest X-ray (*n* = 114)

**Fig. 2:**
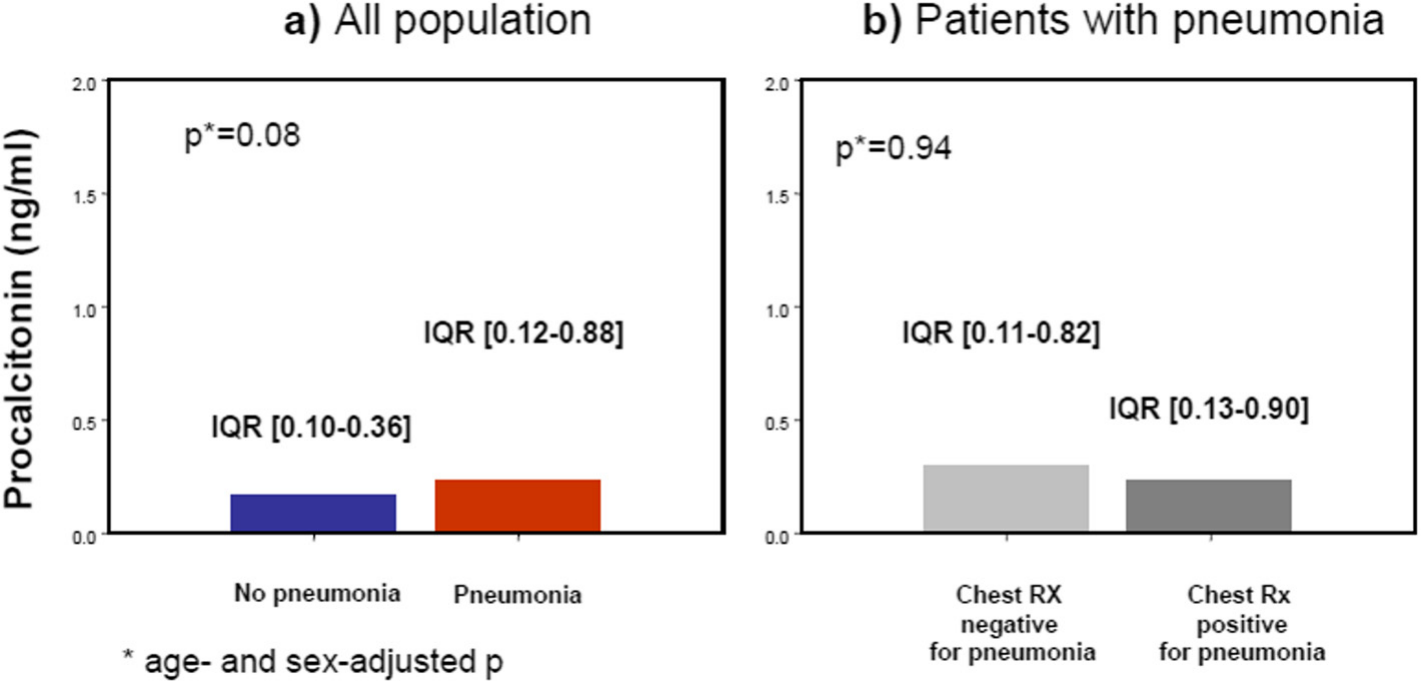
Serum procalcitonin as marker of pneumonia. Panel **a**) Comparison of serum procalcitonin values in patients with (*n* = 239) and without pneumonia (*n* = 216). Panel **b**) Comparison of serum procalcitonin values between patients with pneumonia and a diagnostic chest X-ray (*n* = 125) and patients with pneumonia and a non-diagnostic chest X-ray (*n* = 114)

Serum hs-CRP values at admission were significantly higher in P+ than in P- group (median 116.0, IQR 46.5–179.0, vs 22.5, IQR 6.9–84.4, mg/L, age- and sex-adjusted *p* < 0.001) (Fig. 1), while procalcitonin values were not (median 0.22, IQR 0.12–0.87, vs 0.15, IQR 0.10–0.35, ng/ml, age- and sex-adjusted *p* = 0.08) (Fig. 2).

**Fig. 3:**
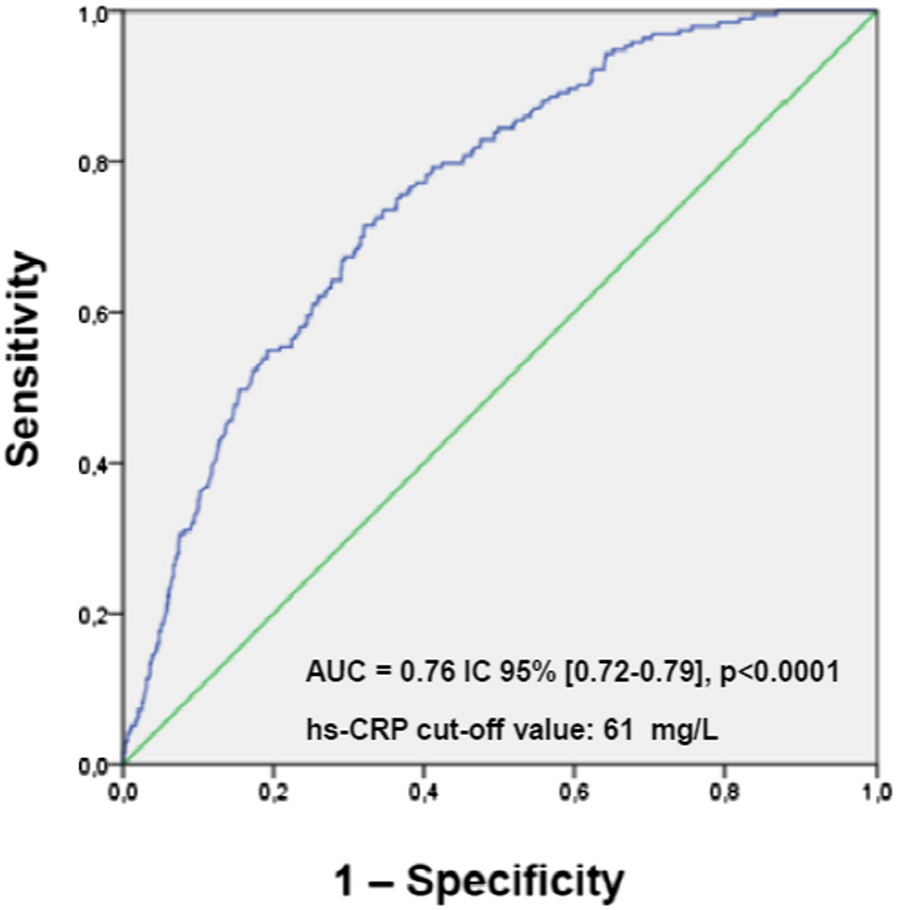
ROC curve of high-sensitivity C-reactive protein (hs-CRP) vs pneumonia diagnosis. ROC curve showing the association of hs-CRP levels at admission with the diagnosis of pneumonia in the studied multimorbid elderly population (AUC 0.76, 95 % CI 0.72–0.79, age- and sex-adjusted *p* < 0.0001, cut-off value 61 mg/L)

The ROC analysis showed that hs-CRP values were significantly associated with pneumonia (AUC 0.76, 95 % CI 0.72–0.79, age- and sex-adjusted *p* < 0.0001, Fig. 3). Inclusion of other covariates that were significantly different between P+ and P- groups (nursing home residency and dementia) in the ROC model did not change this result (AUC 0.78, 95 % CI 0.73–0.81, fully-adjusted *p* < 0.0001). The cut-off value, with the best sensitivity and specificity compromise, was set at 61 mg/L. On the other side, ROC analysis for procalcitonin confirmed that this biomarker is not significantly associated with pneumonia in the studied population (AUC 0.54, 95 % CI 0.47–0.62, age- and sex-adjusted *p* = 0.20).

Univariate (model 1) and multivariate (model 2) Cox regression models were then built to test the association of hs-CRP values, dichotomized according to the ROC method, with pneumonia diagnosis. These models are shown in Table 2. Notably, after adjustment for multiple potential confounders that were significantly different at univariate analysis shown in Table 1, including age, sex, dementia and provenience from nursing home, a serum hs-CRP value >61 mg/L at admission was associated with a 3.59-fold higher risk of pneumonia (OR 3.59, 95 % CI 2.35–5.48, fully-adjusted *p* < 0.0001).

**Table 2:** Multivariate Cox regression models exploring the association between serum hs-CRP >61 mg/L and the diagnosis of pneumonia in the study population (*n* = 455)

Parameter	O.R.	95 % C.I.	*p*
Model 1 (age- and sex-adjusted)			
hs-CRP> 61 mg/L	3.88	2.58–5.81	<0.001
Age	0.98	0.97–1.01	0.17
Sex (F vs M)	0.59	0.39–0.89	0.01
Model 2 (fully-adjusted)			
hs-CRP> 61 mg/L	3.59	2.35–5.48	<0.001
Age	0.98	0.96–1.01	0.09
Sex (F vs M)	0.52	0.34–0.,80	0.003
Setting of living (nursing home vs community)	1.99	1.18–3.36	0.009
Dementia	2.45	1.48–4.08	<0.001

Model 1 is age- and sex-adjusted. Model 2 is adjusted also for covariates that proved significantly different between patients with and without pneumonia at univariate analysis (i.e. dementia and provenience from a nursing home). The cut-off value of hs-CRP was set at 61 mg/L according to the Youden index applied to the ROC method

Among patients with pneumonia, 46 were admitted from a nursing home and therefore had HCAP (21 M, 25 F, mean age 82 ± 14 years), while 193 had been living in the community and were classified as CAPs (117 M, 76 F, mean age 79 ± 13). Neither hs-CRP (median CAP vs HCAP 112.5, IQR 42.5–183.0, vs 127.0, IQR 46.5–176.0, mg/L, age- and sex-adjusted *p* = 0.60) nor procalcitonin values (median CAP vs HCAP 0.22, IQR 0.12–0.79, vs 0.31, IQR 0.12–1.93, ng/ml, age- and sex-adjusted *p* = 0.56) were significantly different between these two subgroups.

Moreover, in patients with pneumonia, CXR was diagnostic only in 125 subjects (52.3 %). Conversely, other 114 patients (47.6 %) had negative or inconclusive radiographic findings and therefore diagnosis was established by chest CT scan. As shown in Figs. 1 and 2, at an age- and sex-adjusted analysis, both hs-CRP and procalcitonin were not significantly different between these two subgroups. Pneumonia was complicated by sepsis, severe sepsis or septic shock only in 11, 10 and 5 cases, respectively (11 % overall).

## Discussion

In a cohort of multimorbid elderly patients acutely hospitalized with respiratory symptoms, serum hs-CRP values >61 mg/L were significantly associated with a diagnosis of pneumonia, irrespective of CAP/HCAP categorization and results of CXR. On the other side, serum procalcitonin levels were not significantly able to discriminate between those patients with and those patients without pneumonia.

Pneumonia in elderly patients may be a real diagnostic challenge, as confirmed by the finding that in our case-series CXR was negative or inconclusive in almost one patient out of two. Many factors, including cognitive impairment, lack of collaboration, low muscle strength and disability, may contribute to this poor diagnostic performance of CXR [27]. Thus, the diagnostic approach should be multimodal, basing on a personalized approach and encompassing not only traditional imaging techniques but also accurate laboratory tests. Unfortunately, no known biomarker holds a sufficient sensitivity and sensibility to be used alone as a valid diagnostic tool for pneumonia, particularly in this setting, where comorbidities may act as strong confounders. This is also the case of hs-CRP and procalcitonin. However, our results strengthen the role of hs-CRP as a guide in the management of acute respiratory symptoms in elderly patients.

In fact, the presence of serum hs-CRP values >61 mg/L in a patient with acute respiratory symptoms strongly suggests the diagnosis of pneumonia. Thus, diagnostic techniques with a higher accuracy for pneumonia than CXR, such as chest CT or, possibly, lung ultrasound, might be performed [28]. On the other side, when hs-CRP is ≤61 mg/L, other diagnostic hypotheses, including COPD exacerbation, asthma and acute heart failure, might be pursued.

Our results instead do not support any diagnostic role of procalcitonin, therefore suggesting that this laboratory test should not be performed routinely in this particular setting.

Up to date, very few studies have assessed the usefulness of these biomarkers for pneumonia diagnosis, focusing exclusively on hospitalized multimorbid elderly. In a recent clinical study from Greece a good diagnostic performance for both CRP and procalcitonin was demonstrated in HCAP [7]. Another prospective study showed that a significant, though late, rise in procalcitonin levels was associated with bacterial infections in elderly inpatients, although this correlation could have poor diagnostic usefulness [17].

In adult patients without significant comorbidities, the measurement of serum procalcitonin is widely recommended as an efficient biomarker of pneumonia, for both diagnostic and prognostic purposes [5–16]. In spite of this, nearly all studies that compared procalcitonin and CRP in pneumonia concluded that the latter exhibits a diagnostic performance that is comparable to, if not better than, that of procalcitonin [7, 9–11, 14, 20]. There is actually only one study clearly concluding that procalcitonin is a better biomarker of pneumonia than CRP [12], while another reported that both tests have no advantages in clinical practice [19].

During the aging process, there may be several elements biasing the association between pneumonia and elevation in serum of CRP or procalcitonin. For example, multimorbid elderly subjects may face complex immunologic rearrangements with chronic exposure to a variety of antigens promoting a subclinical inflammatory status defined as *inflammaging* [29]. These phenomena may result in a peculiar cytokine pattern, with impaired procalcitonin release in response to antigens that generally promote its synthesis from extra-thyroidal sites in adult subjects. Furthermore, the etiology of pneumonia may also be associated with different cytokine activation patterns [30]. Thus, immunologic changes that occur with aging, variability in etiology of pneumonia, multimorbidity and polypharmacy may help explaining why many participants to the present study did not exhibit serum procalcitonin elevation despite a serious infection was present. Moreover, only a small percentage of patients with pneumonia (11 %) developed sepsis, severe sepsis or septic shock. Since procalcitonin elevation is a known marker of sepsis, this may help to explain the relatively low levels of procalcitonin in the studied population.

Our results are consistent with those by van Vugt et al., who found that a CRP >30 mg/L can improve diagnostic classification of patients with CAP in a large cohort of adults referred to primary care facilities [20]. Moreover, some authors have also demonstrated that CRP dosage may be very useful in distinguishing CAP from exacerbations of asthma or COPD, with optimal sensitivity and specificity (respectively 0.91 and 0.93) at a cut-off value of 48 mg/L [10].

These studies identified CRP cut-off values for pneumonia diagnosis lower than that found in our cohort (i.e., 61 mg/L). This difference may be due to the high multimorbidity burden of our patients. Chronic diseases can represent triggers for CRP elevation and possible confounding factors, accounting for the higher median values of inflammatory parameters. Thus, further studies are needed to confirm and validate the detected cut-off value in populations with similar multimorbidity burden.

In our cohort, serum hs-CRP values were not statistically different when comparing patients with CAP and HCAP. A prospective multicenter case–control study carried out in Spain has recently demonstrated that microbial etiology of HCAP is similar to CAP, thus highlighting that this classification may be important only from an epidemiological, and not a clinical, point of view [31]. The role of hs-CRP in pneumonia diagnosis may thus be independent of this classification. However, there are also studies that claim different etiology and clinical course between CAP and HCAP [2]. The different value of biomarkers in CAP and HCAP should therefore be better investigated.

Some limitations should be taken into account when interpreting the results of our study. First, the retrospective design did not allow to collect data about important covariates, such as functional and nutritional status. Thus, further studies with a prospective design are needed to confirm our findings. Second, the etiology of pneumonia was available only in a minority of patients, and thus categorization of results according to this aspect was not possible. Third, no data were available about the number of drugs taken by each patient, particularly immunosuppressive drugs, even if the prevalence of comorbidities that chronically require these medications was very small (below 5 %). However, some evidence suggests that these drugs do not directly influence the concentration of either CRP or procalcitonin, since the variation of serum levels is more strictly associated with the clinical course [32, 33]. Fourth, the elevated clinical complexity of patients due to multimorbidity may also carry out potential biases that cannot be completely measured through CIRS. Finally, stratification of pneumonia severity and prognosis with internationally-validated indexes, like Pneumonia Severity Index (PSI) or CURB-65, was not available for many patients, since it was generally performed in the Emergency Department and not recorded on documents included in our clinical records.

## Conclusions

In a cohort of multimorbid patients aged more than 65 who required hospitalization for acute respiratory symptoms, serum procalcitonin lacks sufficient accuracy for the diagnosis of pneumonia. Therefore, the routine prescription of this laboratory test should be discouraged in this setting. Conversely, hs-CRP elevation over 61 mg/L implies a high risk of pneumonia, and might therefore be useful for clinicians in guiding prescriptions of other diagnostic tests, particularly when CXR findings are inconclusive. Prospective studies focused on this complex population are needed to confirm these findings.

## Abbreviations

CAP: community-acquired pneumonia
HCAP: healthcare-associated pneumonia
CXR: chest x-ray
CRP: C-reactive protein
ICU: intensive care unit
hs-CRP: high sensitivity C-reactive protein
HAP: hospital-acquired pneumonia
CT: computed tomography
CIRS: cumulative illness rating scale
ROC: receiver operating characteristics
AUC: area under the curve
CI: confidence interval
CKD: chronic kidney disease
OR: odds ratio
PSI: pneumonia severity index
